# Structure of a bound peptide phosphonate reveals the mechanism of nocardicin bifunctional thioesterase epimerase-hydrolase half-reactions

**DOI:** 10.1038/s41467-019-11740-6

**Published:** 2019-08-27

**Authors:** Ketan D. Patel, Felipe B. d’Andrea, Nicole M. Gaudelli, Andrew R. Buller, Craig A. Townsend, Andrew M. Gulick

**Affiliations:** 1 Department of Structural Biology, The Jacobs School of Medicine & Biomedical Sciences, State University of New York at Buffalo, 955 Main Street, Buffalo, NY 14203 USA; 2 Department of Chemistry, Johns Hopkins University, 3400 North Charles Street, Baltimore, MD 21218 USA; 3 Present Address: Tri-Institutional MD-PhD Program, Weill Cornell Medical College, 1300 York Ave., New York, NY 10065 USA; 4 Present Address: Beam Therapeutics, 26 Landsdowne Street, Cambridge, MA 02139 USA; 5 Present Address: Department of Chemistry, University of Wisconsin, 1101 University Avenue, Madison, WI 53711 USA

**Keywords:** X-ray crystallography, Biosynthesis, Enzyme mechanisms

## Abstract

Nonribosomal peptide synthetases (NRPSs) underlie the biosynthesis of many natural products that have important medicinal utility. Protection of the NRPS peptide products from proteolysis is critical to these pathways and is often achieved by structural modification, principally the introduction of d-amino acid residues into the elongating peptide. These amino acids are generally formed in situ from their l-stereoisomers by epimerization domains or dual-function condensation/epimerization domains. In singular contrast, the thioesterase domain of nocardicin biosynthesis mediates both the effectively complete l- to d-epimerization of its *C*-terminal amino acid residue (≥100:1) and hydrolytic product release. We report herein high-resolution crystal structures of the nocardicin thioesterase domain in ligand-free form and reacted with a structurally precise fluorophosphonate substrate mimic that identify the complete peptide binding pocket to accommodate both stereoisomers. These structures combined with additional functional studies provide detailed mechanistic insight into this unique dual-function NRPS domain.

## Abstract (web summary)

NocTE is a nonribosomal peptide synthetase thioesterase that completes the biosynthesis of pro-nocardicin G, the precursor for nocardicin β-lactam antibiotics. Here the authors provide mechanistic insights into NocTE by determining its crystal structures in the ligand-free form and covalently linked to a fluorophosphonate substrate mimic.

## Introduction

Many important natural products are produced by nonribosomal peptide synthetases (NRPSs), a family of enzymes that deploys a modular biosynthetic strategy to produce diverse molecules critical to bacterial physiology and pathogenesis^1^. Each module contains a set of catalytic domains and a peptidyl carrier protein (PCP), often joined on a single polypeptide chain^2^. The PCP is post-translationally modified with a coenzyme A-derived phosphopantetheine cofactor that serves as a transient linker on which both amino acid building blocks and the extending peptide chain are covalently bound as thioesters during synthesis^3^. The domains are organized into modules that maintain the necessary activities to activate and incorporate a single amino acid substrate as well as form a peptide bond, two core reactions that are catalyzed by an adenylation (A) and condensation (C) domain, respectively. Because the nascent peptide is covalently attached to the biosynthetic machinery, a release step is required that is generally catalyzed by a thioesterase (TE) or reductase domain.

While the canonical biosynthesis carried out by NRPSs has been well-studied, an increasing number of examples exist in which NRPS domains are shown to catalyze unusual activities^4^. These activities include amide formation by adenylation domains^5,6^, condensation domain-catalyzed amide formation between a propionate and a pteridine ring in an unusual “pepteridine” natural product^7^, thioesterase domain-mediated condensation of two α-keto carboxylates in the production of quinone or furanone derivatives^8,9^, β-lactone and β-lactam cyclization by thioesterase domains^10–12^, and a Dieckmann condensation catalyzed by an unusual reductase domain, which lacks the conventional catalytic triad, involved in the release of cyclopiazonate tetramic acid neurotoxin^13^. Some of these activities reflect more limited changes in the overall synthetic program, for example, the use of an alternate nucleophile to attack the adenylate directly in formation of the amide bond that bypasses the PCP-bound thioester intermediate. Curiously, however, some NRPS domains catalyze chemical reactions that had never been functionally assigned to related domains.

**Fig. 1:**
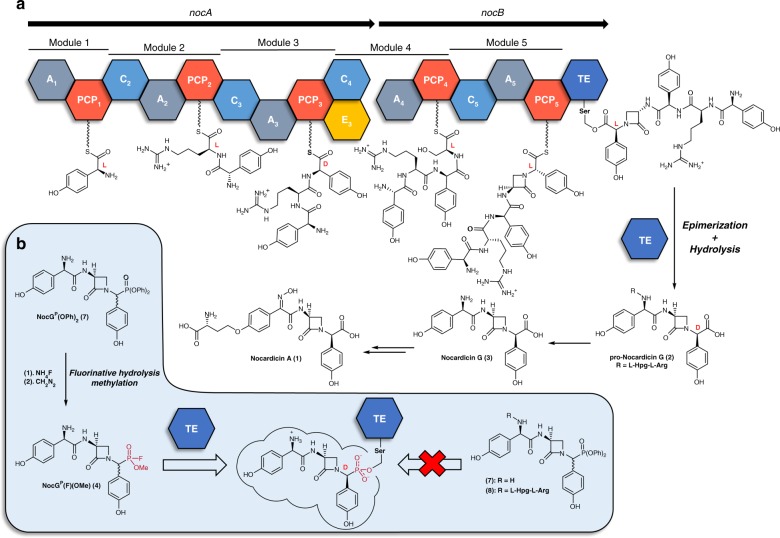
Nocardicin biosynthetic pathway. **a** Nocardicin biosynthesis is initiated through the activities of five modules spanning two proteins, NocA and NocB. The final steps in the release of the pro-nocardicin G pentapeptide are carried out by the bifunctional thioesterase domain (NocTE), which catalyzes both epimerization of the C-terminal Hpg residue and hydrolysis. **b** Whereas diphenylphosphonate analogs did not react with the catalytic nucleophile of the thioesterase domain, a fluorophosphonate tripeptide analog **4** inactivated the enzyme and was used in structural studies to characterize the active site

The NRPS enzymes that produce the β-lactam antibiotic nocardicin A (**1**) exhibit several unusual features (Fig. 1). The importance of β-lactam antibiotics and the marked diversity of biosynthetic approaches to the members of this natural product family have made them attractive for study^14^. The nocardicin A core is ultimately derived from three amino acids, two nonproteinogenic D-(*p*-hydroxyphenyl)glycine (D-Hpg) residues surrounding a seryl residue-derived lactam ring. Contrary to the expectation that the tripeptide precursor would be produced by a three-module system, pro-nocardicin G (**2**) is produced through the combined action of NocA and NocB, two NRPSs that provide a total of five modules. Nocardicin G (**3**), the simplest of the nocardicins and the precursor of nocardicin A, is derived from the residues at positions 3–5 after the late removal of the N-terminal two residues by an unknown cellular protease. Further, β-lactam formation was shown to be catalyzed by the condensation (C) domain of the fifth module, using an unusual **H**HHxxxDG motif in place of the highly conserved HHxxxDG signature of most condensation domains^15,16^. Finally, despite having only a single epimerization domain, which resides within the third module, nocardicin A contains two D-Hpg residues at the third and fifth position of pro-nocardicin G. Studies to determine if the epimerization domain acted on substrates bound to alternate modules led to the discovery that the terminal thioesterase domain (NocTE) exhibited unprecedented epimerase activity to produce the C-terminal D-Hpg^17^.

Careful kinetic analysis to elaborate the steps of nocardicin production with multiple peptide analogs demonstrated the remarkable selectivity of the NocTE domain for peptides containing the β-lactam. At rates comparable to controls, the NocTE very slowly hydrolyzed *N*-acetylcysteamine (SNAC) peptides containing a serine or alanine in position four regardless of the stereochemistry in the fifth Hpg residue. In contrast, SNAC peptides containing the β-lactam in the fourth position rapidly reacted. These studies further showed that the epimerization is catalyzed by the TE domain after transesterification of the peptide to the serine of the NocTE catalytic triad and not as a PCP-bound thioester. Finally, comparison of the rates of reaction of the peptides containing D-Hpg and L-Hpg showed that epimerization is partially rate-limiting, occurring at a rate ~10× slower than hydrolysis^17^.

To complement and further interpret a body of mechanistic studies^10,15,17,18^ to understand the unusual steps in nocardicin A biosynthesis, we present structures of this dual-function thioesterase domain NocTE in the unliganded and peptide-bound states. Structural studies of NRPS catalytic domains benefit from the availability of ligands or ligand analogs to provide insight into the active site architecture and catalytic mechanism^19^. We have, therefore, designed and prepared a fluorophosphonate (FP, **4**) analog of nocardicin G (**3**) that captures an accurate substrate acyl-enzyme mimic of the final tetrahedral transition state leading to product release and provides an intimate view of the complete peptide substrate not previously observed in an NRPS TE domain active site. Analysis of the data identifies a two-part binding pocket that is likely used for the C-terminal Hpg in each of its diastereomeric forms during epimerization as well as supporting involvement of the core catalytic triad in the epimerization step. These residues were probed using site-directed mutagenesis and functional analysis of both steps catalyzed by NocTE.

## Results

### The structure of NocTE

**Fig. 2:**
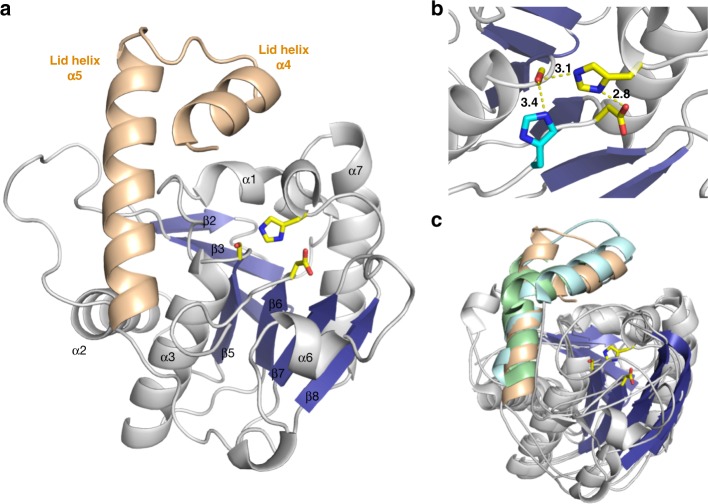
Structure of NocTE. NocTE forms a traditional α/β hydrolase fold observed with NRPS thioesterase domains. **a** The lid helices α4 and α5 are highlighted in wheat. The catalytic triad residues Ser1790, His1901, and Asp1806 are shown in yellow. In the absence of ligands, the N-terminal portion of helix α4 is disordered. **b** Catalytic triad residues are highlighted in yellow, while one side-chain orientation of His1808 is shown in cyan. **c** Superposition of PksA TE (PDB 3ILS) and Srf TE (2VSQ) with NocTE, showing similar lid helix positions. The lid helices of PksA are colored cyan and the helices of SrfA-C are colored green. Loop smoothing is used in (**c**) for clarity

Previous experiments had demonstrated that the epimerization and thioester cleavage reactions take place on β-lactam containing peptide substrates bound to Ser1779 and not while still linked to a PCP, as observed for E domains^17^. To confirm bioinformatic assignment of the catalytic triad, guide mutagenesis experiments and identify additional residues that could play catalytic roles, the structure of NocTE was solved by MAD phasing and subsequently refined against a high-resolution dataset from a native protein crystal. The overall structure of NocTE is similar to known TE domain structures of NRPS and polyketide synthase (PKS) enzymes (Fig. 2)^20^. A search for the closest structural homologs with the DALI server^21^ indicated that NRPS TE domains from surfactin (PDB 1JMK)^22^ and enterobactin (PDB 5JA2)^23^ biosynthesis, the PKS TE domain from aflatoxin biosynthesis (PDB 3ILS)^24^ and the type-II TE domain RifR (PDB 3FLA)^25^ are closest to the NocTE domain with rms displacements of 2.3–3.0 Å. The typical α/β hydrolase fold consists of a central seven stranded β-sheet (β2–β8) surrounded by two and three helices on either side, while two helices on top form the lid region (α4 and α5). Like other TE domains of NRPS and PKS origin, the first N-terminal β-strand of the hydrolase fold is not present in the NocTE domain. The second β-strand is the only anti-parallel strand of the remaining six strands in the structure. The lid region of NocTE consists of two helices (α4 and α5) that are oriented at an angle of 48° relative to each other and are held together through hydrophobic interactions. The α4 helix of the lid is furthest away from the active site (Fig. 2) and makes minimal contacts with the core of the domain. In contrast, the second lid helix α5 makes extensive contacts with the core through interactions with the α2 and α3 helices and the loops that follow them. The observed positions of the NocTE lid helices are similar to the PksA TE (PDB 3ILS) and Srf TE (PDB 2VSQ)^26^, as shown in Fig. 2c.

The NocTE active site structure with its catalytic triad Ser1779, His1901, and Asp1806 is similar to canonical Type-I thioesterase domains (Fig. 2a). The nucleophilic Ser1779 belonging to the GXSXG motif is present on the loop following the β5 strand and His1901 is located on the loop after β8. The acidic residue Asp1806 is positioned according to its canonical site in α/β hydrolase folds at the loop following the β7 strand. The residues of the catalytic triad form a typical hydrogen bonding network. Interestingly, positioned two residues downstream from the catalytic aspartate (Asp1806) is His1808, which forms an additional hydrogen bond with the catalytic serine (Fig. 2b). In the unliganded structure, His1808 adopts two alternate side chain positions; the principal orientation hydrogen bonds to Ser1779.

### Site-directed mutagenesis of catalytic residues

The crystal structure of NocTE confirmed the assignment of His1901 and Asp1806 in the catalytic triad with Ser1779, and additionally identified potential catalytic involvement by His1808. Given its location in the active site and lack of conservation in canonical thioesterase domains, we used site-directed mutagenesis to examine the role of these residues in the unusual epimerization–hydrolysis steps mediated by NocTE.

**Fig. 3:**
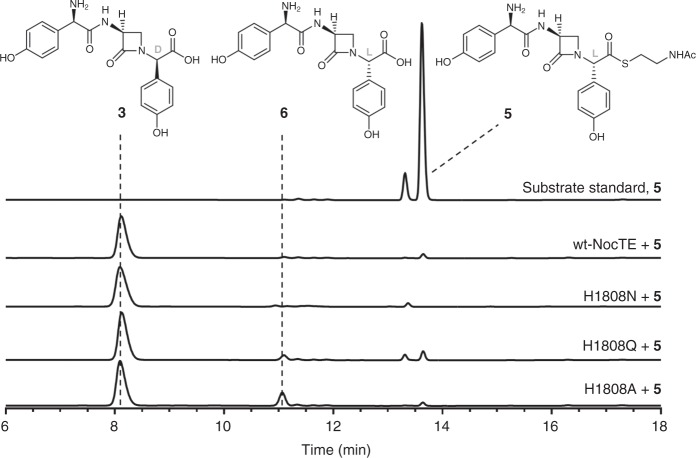
Mutational analysis of His1808. Wild-type or mutant NocTE was incubated with the SNAC thioester of the *epi*-nocardicin G **5** and monitored by HPLC for its ability to catalyze epimerization and hydrolysis to **3** or hydrolysis to **6**. Chromatograms for assays were monitored at 272 nm. Mutation of His1808 had little impact on catalytic activity, but the fraction of **6** in the product rises from ≤1% in wild type to ~15% in the H1808A mutant

Previous work had established that mutation of the catalytic serine to alanine completely abolished activity^17^. Site-specific mutants of the remaining residues His1901 and Asp1806 to alanine were individually constructed and assayed^17^ against the *N*-acetylcysteamine thioester (SNAC) of *epi*-nocardicin G (**5**, Fig. 3). While the His1901 mutant was completely devoid of detectable activity, substitution of the catalytic aspartate with alanine resulted in full epimerization and hydrolysis, but at a rate lower than the wild type enzyme (Supplementary Fig. 1). Three different variants of NocTE were then prepared to investigate what role, if any, the “extra” histidine residue, His1808, might have on the dual function TE. This histidine was replaced by alanine, glutamine, and asparagine where the latter were intended to mimic the corresponding ε- and δ-imidazole nitrogens of histidine. As depicted in Fig. 3, mutation had little effect on the epimerization/hydrolysis reaction showing only some degradation of stereochemical control in the appearance of *epi*-nocardicin G (**6**) in the product profile. Even complete removal of the His1808 side chain in the H1808A mutant resulted in a protein that was largely still active. Thus, it appears that His1808 plays no catalytic role. However, given the increased proportion of **6** in the assays with the mutant enzymes of decreased steric size, His1808 may help enforce substrate orientation in the active site of NocTE. It is to be noted that, although the SNAC thioesters of nocardicin G (**3**) and *epi*-nocardicin G (**5/6**) could be synthesized in stereochemically pure form in dry, organic solvent, upon addition to aqueous assay buffer spontaneous *C*-terminal epimerization takes place with a half-life of ~21 min^27^. Notwithstanding this comparatively rapid rate as evident in the HPLC trace of substrate **5** in Fig. 3, the combined NocTE-catalyzed epimerization and hydrolysis occurs ~1200 times faster^17^.

### Inhibitor design and analysis

Due to the natural hydrolytic or macrocyclization activity of NRPS thioesterase domains, it has proved challenging to obtain structures of acyl-enzyme intermediates. To best explore the structural and mechanistic features of the unique epimerase/hydrolase chemistry performed by NocTE, we pursued the design of small-molecule inhibitors capable of producing an enzyme-inactivator adduct with maximal native structural fidelity^28^. Biochemical characterization of NocTE had demonstrated its marked selectivity towards β-lactam-bearing, tri- and pentapeptide *N*-acetylcysteamine (SNAC) thioester substrate analogues^17^. As a consequence, candidate inhibitors must retain key elements like the azetidinone ring, appropriate amino acid sequence, and stereochemistry to meaningfully duplicate interactions occurring within the NocTE active site.

We first pursued a diphenylphosphonate (DPP) warhead not only because of its well-documented success as a seryl-reactive group, but also because of the ease with which it could be incorporated into amino acid analogues^29–31^. A peptide analog that employed other serine hydrolase inhibitor classes would either require more complicated syntheses (e.g., boronic acids^32^), introduce unwanted bulk into the active site (e.g., trifluoromethyl ketones^33,34^), or be incompatible with the Hpg moiety by preliminary experiments (e.g., aldehydes^35^). Despite successfully synthesizing both tripeptide nocardicin G and pentapeptide pro-nocardicin G DPP analogues **7** and **8** (Fig. 1), respectively, incubation with NocTE in a variety of conditions did not result in any detectable adducts by mass spectrometry. Ultimately, the serendipitously discovered ability to convert the DPP group to a much more reactive fluorophosphonate (FP) warhead (**4**, Fig. 1) using a late-stage, two-step fluorinative hydrolysis and methylation protocol led to a potent and selective inactivator of NocTE that was stable in buffer for prolonged periods of time and conserved the desired structural properties of the enzyme^28^.

### Structure of NocTE bound to tripeptide inhibitor

**Fig. 4:**
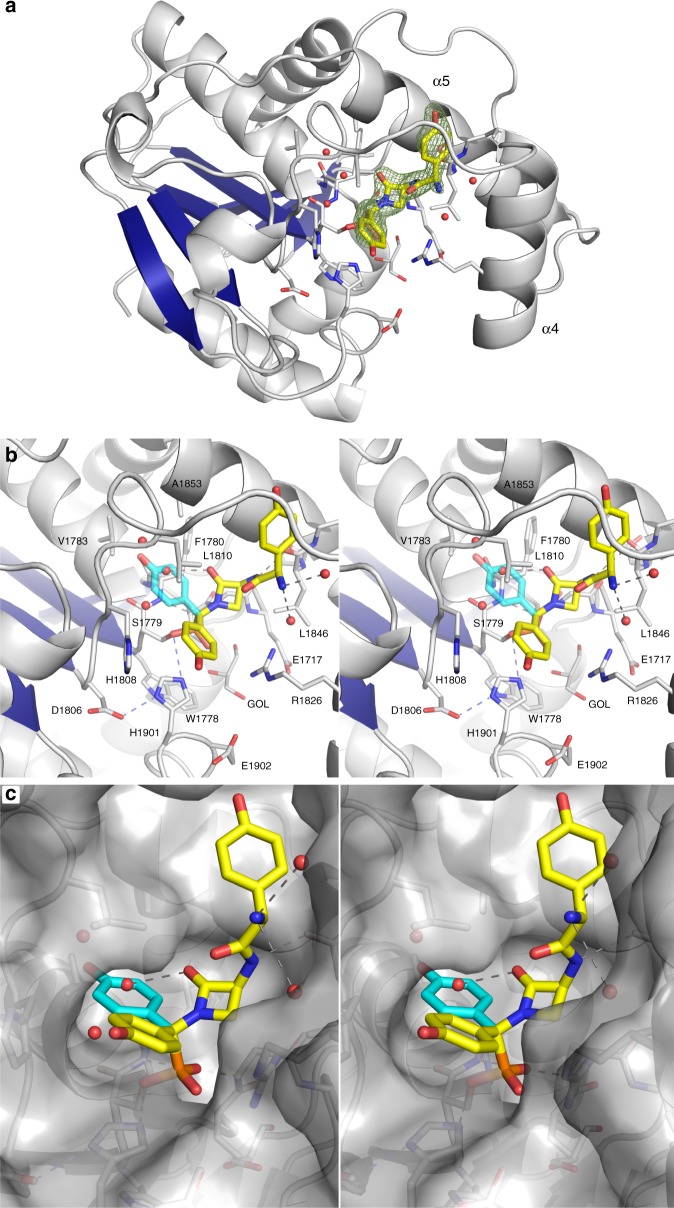
The structure of NocTE reacted with fluorophosphonate **4**. **a** Cartoon representation of NocTE. The central sheet of the protein is highlighted with blue. The ligand is shown with yellow carbon atoms. A glycerol molecule that co-crystallized in all four chains is shown at the base of the pocket. Simulated annealing omit map electron density calculated is shown for the peptide ligand. **b** Stereorepresentation of the active site. The captured ligand in the D-configuration is shown with yellow carbon atoms. Superimposed on the structure in cyan is the alternate L-stereoisomer of the C-terminal HPG residue, which was manually docked into the structure. In this orientation, the phenyl moiety occupies a hydrophobic cavity formed by residues Val1783, Ala1853, Phe1780, and Leu1810 in the NocTE structure. Several waters occupy this pocket in all chains of the structure, with one water molecule potentially approximating the location of the L-Hpg hydroxyl. One oxygen atom from the phosphonate projects back towards the oxyanion hole formed by the amide nitrogens of Phe1780 and Gly1716. Hydrogen bonds are shown in gray, with those of the catalytic triad shown in blue. **c** Surface representation of NocTE highlights the cavity for binding the substrate L-Hpg (cyan) as well as the more open pocket for the D-Hpg (yellow) product

Initial attempts to introduce the covalent ligand into pregrown crystals by soaking proved unsuccessful. We, therefore, identified new crystal growth conditions of the covalently modified NocTE accessed by preincubation with FP **4** (Fig. 4). The covalently modified NocTE crystallized in space group P2_1_2_1_2_1_ with four protein chains in the asymmetric unit. The structure of NocTE bound to ligand showed excellent density (Supplementary Fig. 2) for the covalent modification of Ser1779 in all four chains. The density was unequivocal for the complete ligand in chains A and B. Electron density was of lower quality in chains C and D. A glycerol molecule from the cryoprotection solution is located between the phosphonate moiety and bulk solvent in three chains. No density was observed in any of the four chains for a methyl group attached to the phosphonate of the ligand, likely owing to hydrolysis, or well-precedented “aging”, during the extended period of crystal growth^36^.

Phosphonate-based inactivators have been successfully used to crystallize acyl-enzyme complexes and mimic tetrahedral intermediates of serine protease^36^ as well as PKS TE domains^29^. The NocTE-complex structure with the “aged” phosphonate–Ser adduct (loss of methanol) mimics closely the tetrahedral covalent intermediate of the hydrolysis half-reaction (Fig. 4). Unlike the dynamic lid helices of TE domains from Srf TE^22^ and Vlm2^37^, NocTE did not show any major rearrangements after ligand binding. Instead, ligand binding caused two minor perturbations in the lid α4 helix (Supplementary Fig. 3). In the unliganded structure, the only contact made by the α4 helix to the core domain is through the backbone carbonyl of the Glu1829 to Arg1903 side chains. These contacts are lost upon ligand binding as the α4 helix has moved further away from active site and the Arg1903 side chain is shifted outwards. Second, ligand binding accompanies an ordering of the N-terminal turn of the helix and the preceding loop. This perturbation enables the formation of a partial hydrophobic groove for interaction with the N-terminal D-Hpg. Since residues from two lid helices and the loop preceding the α4 helix make intricate hydrophobic interactions with the N-terminal Hpg, the lid in NocTE appears to be involved in determining specificity for the aryl residue at this locus in the substrate.

### Substrate interacting residues

The inhibitor is bound into the substrate-binding pocket of the TE domain (Fig. 4). The N-terminal Hpg group sits in a hydrophobic groove formed by Pro1819, Val1823, Leu1847, Gly1850, and Ala1854. The planar β-lactam ring is positioned at the center of the active site cavity and makes hydrophobic interaction through C_β_ with Leu1846 and Gly1716 (Supplementary Fig. 4). The inhibitor cradles the Leu1810 side chain with both Hpg moieties making hydrophobic interactions. The C-terminal Hpg group points toward an exit channel and makes distal hydrophobic interactions with His1808 and catalytic His1901. The His1808 side chain has fully rotated away from the active site to accommodate positioning of the C-terminal Hpg group. Arg1826 is drawn toward the phosphonate group of the inhibitor, making a hydrogen bond with the second phospho-oxygen and additionally makes hydrogen bonds with the side chain of the catalytic Asp1806. The guanidinium of Arg1826 also forms one side of the pocket in which the C-terminal L-Hpg resides.

The backbone amides of Gly1716 and Phe1780 are in hydrogen bonding distances of 2.7 and 2.9 Å to one phospho-oxygen of the ligand and form the oxyanion hole (Supplementary Fig. 5). Catalytic His1901 is positioned to hydrogen bond with the second phospho-oxygen group, indicating that this oxygen may mimic the attacking water molecule in the post-epimerization hydrolysis reaction (see below). Coordination by the histidine residue from the catalytic triad serves to activate the water for attack during the hydrolysis of the epimerized peptide.

The fluorophosphonate probe **4** was incubated with NocTE as an epimeric mixture at the Hpg C-terminus. The final ligand density clearly shows the presence of only D-Hpg in the structure. (We note that, because of the replacement of the carbonyl with the phosphonate, the formally defined *R/S* stereochemistry of the peptide and the FP probe at the C-terminal residue are inverted. We will refer to the observed orientation as the D-epimer, reflecting the configuration of the peptide and not the probe.) Thus, NocTE either selected the D-Hpg diastereomer from the reaction mixture or bound to both stereoisomers and catalyzed the conversion of L-Hpg to D-Hpg at the enzyme active site. The resulting seryl-phosphonate adduct subsequently withstood hydrolysis resulting in the observation of only the peptide harboring the D-epimer. NocTE possesses a large hydrophobic pocket in the active site capable of accommodating the L-Hpg C-terminal epimer. This pocket is formed by the side chains of Val1783, Leu1810, Ala1853, and the main chain of Cα of Phe1780 (Fig. 4b). Alignment of TE domain sequences from 10 homologs that share 57–100% sequence identity shows that the catalytic triad is completely conserved. Additionally, there is strong homology in the other substrate-binding residues described in Fig. 4 with a few conservative changes of Val1783 to Thr, Pro1819 to Ala, and Arg1826 to His. Only Leu1847, which is near the N-terminal D-Hpg but points away from the active site shows more significant variation, appearing as a glutamic acid in several homologs.

**Fig. 5:**
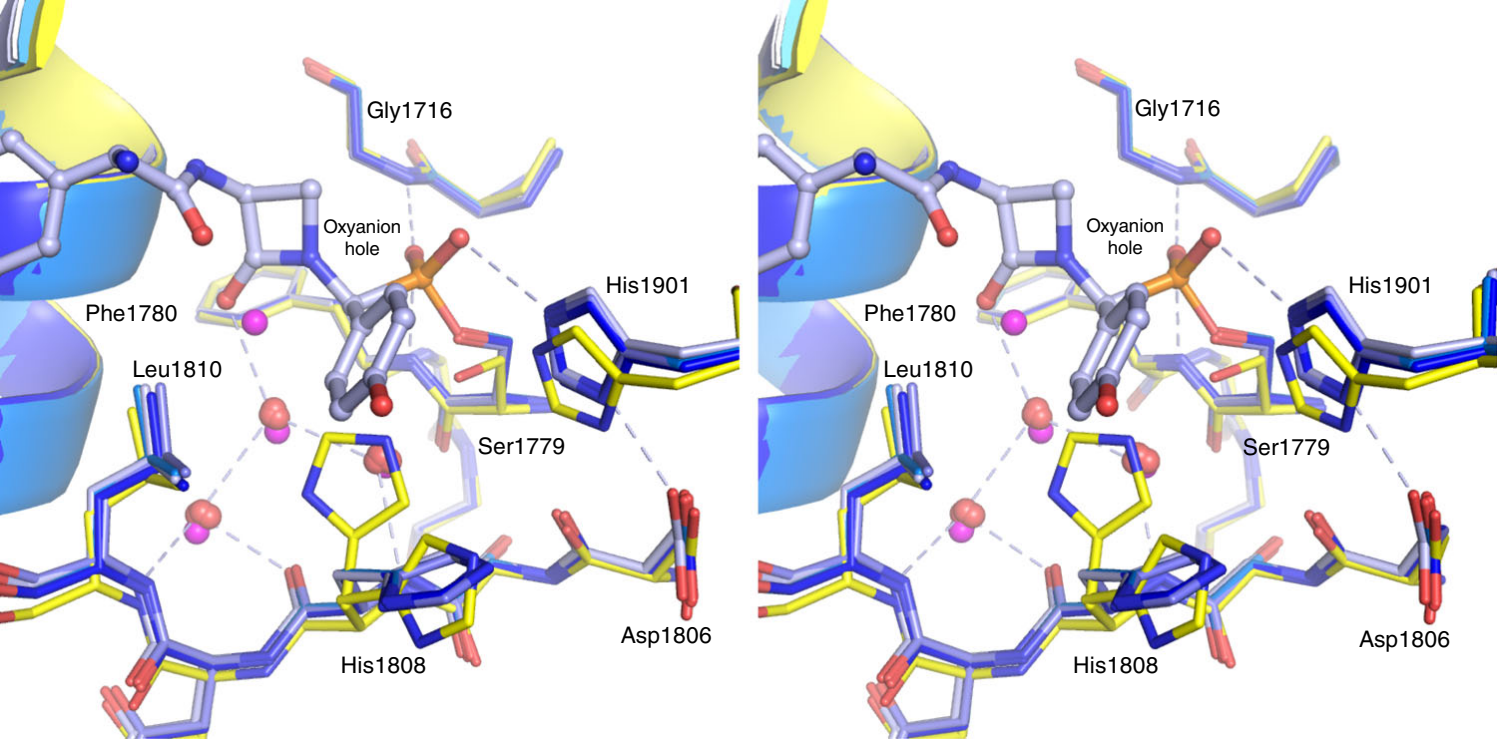
Water network of the NocTE active site. Four chains of the inactivated NocTE (shown in different shades of blue) were superimposed on unliganded NocTE (yellow). Water molecules that fill the putative L-Hpg pocket are shown as spheres from the liganded (red) and unliganded (pink) structures. The side chain of His1808 adopts alternate conformations in the unliganded structure. The covalent adduct formed by reaction with inactivator **4** is shown in ball-and-stick from chain A only. Hydrogen bonds that form the network of interactions among the conserved waters, as well as for the catalytic triad and the oxyanion hole, are highlighted for chain A

We superimposed the four independent chains of NocTE bound to FP **4** on the unliganded model. The structures of the active site, including the water network, are remarkably conserved (Fig. 5). Three water molecules are conserved in the putative L-Hpg pocket. These waters form a network of hydrogen bonds. In the unliganded structure, a fourth water is present that is positioned near the β-lactam carbonyl oxygen. One of these waters approximates the *p*-OH group and interacts with the amide of Leu1810 and the carbonyl oxygen of His1808, suggesting these main chain atoms may coordinate the phenolic hydroxyl of the L-Hpg isoform. As noted above, His1808 adopts two conformations in the unliganded structure. In contrast, in all four chains of the NocTE bound to **4**, this residue adopts a single conformation in which the side chain has cleared the active site making room for the D-Hpg side chain. The water molecules in the L-Hpg pocket are nearly identical in the unliganded and the D-Hpg bound states. These water molecules presumably must vacate this space when the L-Hpg peptide binds, prior to epimerization.

### Comparison with other TE domain structures

**Fig. 6:**
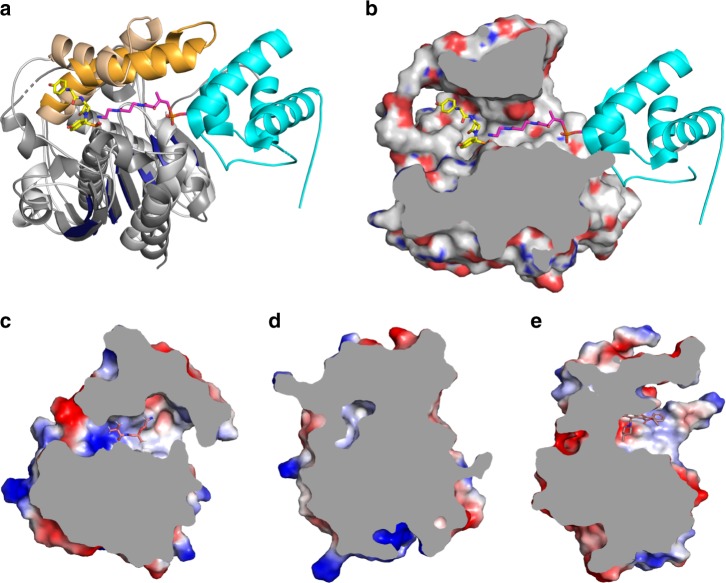
Ligand binding pockets of NocTE and other TE domains. **a** Superposition of the EntF PCP-TE di-domain (PDB 3TEJ) on NocTE. The core N-terminal α/β hydrolase fold is colored gray and deep blue, lid helices in orange and wheat respectively. The EntF PCP domain is colored cyan and pantetheine arm in magenta. The pantetheine atoms derived from the 3TEJ, substrate analog are shown in magenta. The pantetheine terminates in a nitrogen derived from the amide analog used in this study. The NocTE inhibitor is in yellow, blue and red elemental colors. **b** Superposition of EntF PCP-TE di-domain on NocTE showing PCP-docking site and pantetheine tunnel in NocTE electrostatic surface representation. **c** Channel open at two-ends in TE domain in Vlm2 (6ECE). **d** Closed channel with a cavity at catalytic site in PksA TE domain (3ILS). **e** One-end open channel in Pks13 TE domain (5V3X)

We compared NocTE with the didomain PCP-TE structure of EntF^38^ to identify the pantetheine binding site (Fig. 6). The PCP docking site is fairly open and is followed by a tunnel from where the phosphopantetheine arm can deliver the substrate to the catalytic Ser1779. The NocTE substrate channel is centered at a deep crevice where the catalytic nucleophile is positioned and opens to the wide exit site. The shape and orientation of the binding pocket of different TE domains are distinct, with some domains possessing an open channel, while the pocket for other TE domains is more closed (Supplementary Fig. 6). The NocTE active site is a fairly open channel allowing entry of solvent molecules at the active site as revealed in the NocTE unliganded structure. The dynamics of the lid helices are further reflected by the fact that the pockets in some of the unliganded structure collapse to appear almost completely closed in the crystal structures.

The loop joining the β6 strand and α4 lid helix, residues Arg1815–Glu1821, is missing in the NocTE unliganded structure. Upon ligand binding, this loop becomes ordered, although it adopts modestly different positions in the four chains. The NocTE-complex showed the lid helix α5 interacting with the N-terminal D-Hpg of the ligand (Supplementary Fig. 4). Similar intricate interactions are observed with corresponding lid helix in *M. tuberculosis* Pks13 TE domain with a non-covalent benzofuran inhibitor and in PikTE domain with macrolactone product 10-deoxymethynolide and affinity labels (Supplementary Fig. 7). A common theme emerges where lid helix α5 may be important for ligand interactions in other TE domains with similar lid helices.

**Fig. 7:**
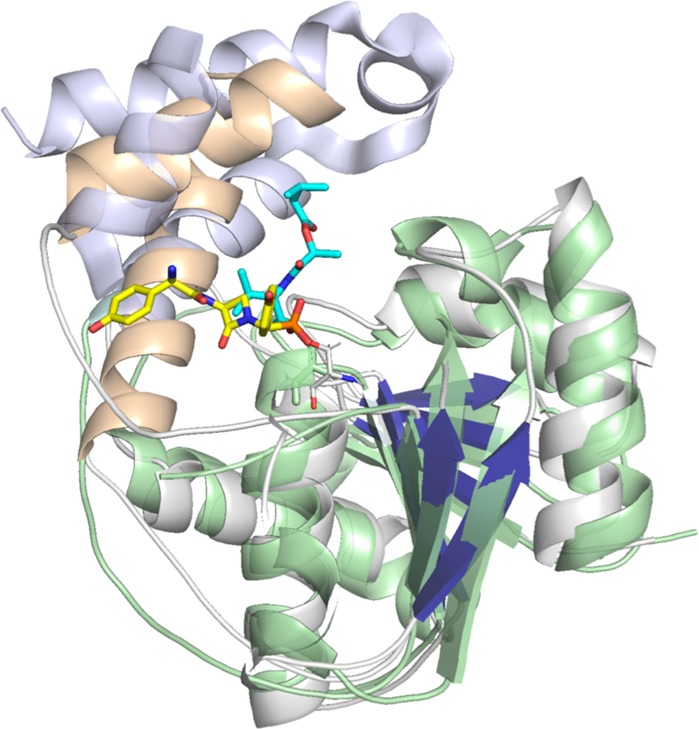
Superposition of NocTE with VlmTE. Distinct orientations of the ligands are shown with the FP probe **4** directly binding between lid helices α4 and α5, while the VlmTE depsipeptide adopts a different orientation that may facilitate the return of the peptide for macrocyclization

We compared the position of the ligand in NocTE to the recently determined structure of the valinomycin TE that utilized a 1,3-diaminopropionate non-coding amino acid to capture a portion of the ligand bound to the catalytic serine^37^. The VlmTE contains a much more extensive lid region of six α-helices, Lα1 through Lα6 (Fig. 7). In the VlmTE structure, helices Lα1 and Lα5 approximate the positions of NocTE helices α4 and α5, respectively, although Lα1 runs antiparallel to NocTE α4. Compared to the four ordered residues of the depsipeptide covalently bound to VlmTE observed in several chains, the nocardicin peptide adopts a distinct position, such that the N-terminal D-Hpg is sandwiched between the two lid helices. Other TE domains may therefore bind their peptides into a similar pocket as the N-terminus of the peptide does not need to access the thioester bond for cyclization.

## Discussion

The appearance of D-amino acid residues in peptides and peptide natural products confers proteolytic stability as well as functional and structural diversity. Inversion of stereochemistry typically owes to racemases or peptidyl epimerases that generate an equilibrating pool of L- and D-antipodes or diastereomers, respectively, from which the D-configured substrate is selectively processed by a downstream enzyme^39^. Additionally, some NRPS systems have integrated epimerization domains within the NRPS assembly line architecture. These epimerization domains are structurally similar to the NRPS condensation domain and likely are evolutionarily related^40^. Lanthipeptides, a subset of ribosomally synthesized and post-translationally modified (RIPPs) natural products can contain D-alanine and D-ethylglycine residues, which arise correspondingly from L-Ser and L-Thr in a specialized two-step elimination/reduction process^41,42^. More recently irreversible L →  D epimerizations have been discovered at multiple sites mediated by single radical *S*-adenosyl-L-methionine (rSAM) enzymes^43,44^. In contrast, NocTE is a unique example where a thioesterase domain catalyzes a functionally unidirectional L → D epimerization that expands the known repertoire in nature to achieve this simple but powerful modification.

**Fig. 8:**
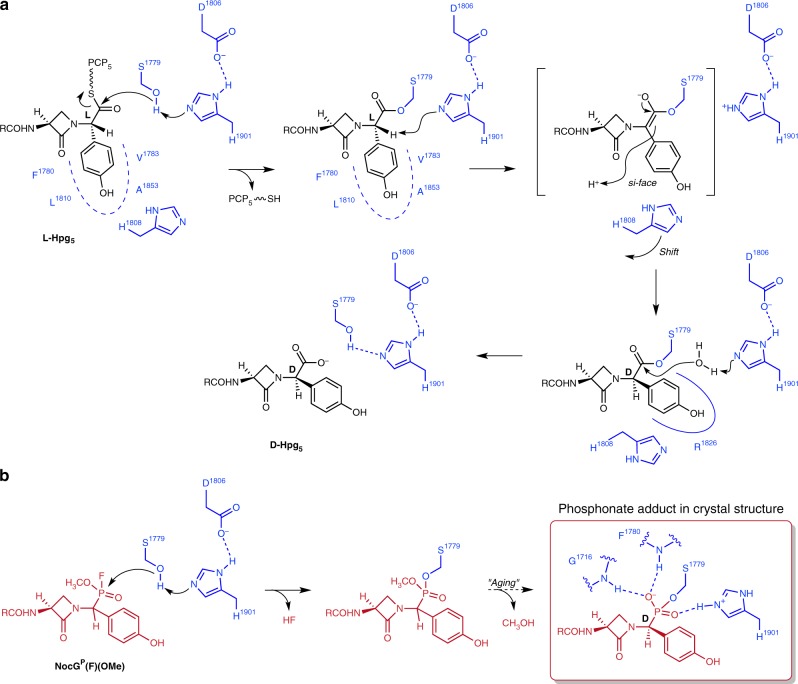
Proposed NocTE catalytic mechanism. **a** The proposed sequence of transthiolation, epimerization, and hydrolysis steps catalyzed by NocTE is depicted. The residues that comprise the binding pockets for the L-Hpg and D-Hpg are indicated with the dashed and solid curves, respectively. The movement of the His1808 side chain that accompanies epimerization is also represented. **b** Illustration of attack and aging of the FP probe. The boxed structure is observed in the crystal and represents the species directly above it in (**a**), highlighting how the phosphonate oxygens mimic the oxyanion hole and the attacking water molecule in the hydrolysis step

The question arises as to the identity of the catalytic residues specifically involved in the conversion of the C-terminal L-Hpg to D-Hpg within the thioesterase domain. We propose that the triad residue His1901 additionally fulfills this role (Fig. 8). The imidazole nitrogen is located ~4.5 Å from the Cα position of D-Hpg in the final model and is positioned with appropriate geometry for abstraction of the α-hydrogen from the L-Hpg substrate. The resulting carbanion, which is planarized and stabilized by delocalization into the *p*-hydroxyphenyl ring, may then be reprotonated from the opposite side to invert the stereochemistry to D-Hpg (Fig. 5a). There are no obvious proton donors apart from water located near the face opening into the hydrophobic pocket proposed for the L-Hpg aryl ring. This lack of a specific proton donor implies that this cavity also lacks a proton acceptor (base) that would efficiently catalyze the formation of L-Hpg if the enzyme is provided with a D-Hpg peptide.

The kinetic behavior of C-terminal epimerization and hydrolysis half-reactions of NocTE have been examined and the latter is ~10 times faster than the former^17^. Kinetic resolution by the thioesterase step, therefore, does not fully account for the ≥100:1 diastereomeric purity of the product. The epimerization step, however, is helped in this instance by the acidity of the α-position of the seryl-bound Hpg unit. Its benzylic nature results in a pKa dramatically lower than other peptide residues. It is also known that NocTE is acutely selective for the β-lactam containing peptide over its corresponding D,L,L- and D,L,D-tripeptides where the embedded β-lactam has been replaced by its precursor, L-serine, or the smaller, hydrophobic L-alanine to mimic the β-lactam ring^17^. The D,L,L-tripeptide β-lactam as its *N*-acetylcysteamine thioester **5** has been determined to bind to NocTE, which then catalyzes C-terminal epimerization and hydrolysis to release nocardicin G (**3**)^17^. We propose that an empty binding pocket evident in the NocTE-complex structure accepts the L-Hpg hydroxyphenyl ring and positions the α-hydrogen close to His1901 of the catalytic triad for deprotonation. For NocTE, deprotonation takes place in preference to conventional water activation and hydrolysis that would release *epi*-nocardicin G (**6**). Consistent with the incorporation of heavy isotope from D_2_O medium^17^, protonation from the opposite face and the absence of a base other than water to mediate kinetically competitive reversion to the D,L,L-diastereomer, comparatively rapid hydrolysis then accounts for the functionally complete epimerization. The C-terminal Hpg after epimerization occupies a second, perhaps thermodynamically favored, hydroxyphenyl binding site observed in the crystal structure, which may further contribute to the processivity of this dual-function enzyme. The transition state of the final hydrolysis event is trapped in the crystal structure of the phosphonate inactivator bound at the catalytic serine residue and defines the oxyanion hole common to all transacylation steps.

While many TE structures have been determined, few bear bound substrates or structurally accurate inhibitor mimics. The discovery^28^ of a mild, two-step method to convert diphenylphosphonate **7** to the more reactive fluorophosphonate **4** enabled an exceptional view into the dual functions of NocTE at high resolution. A complementary approach to characterize the acyl-enzyme intermediate has recently been described that utilizes genetic code expansion to incorporate a 1,3-diaminopropionate non-canonical amino acid in place of the catalytic serine^37^. This protein engineering strategy replaces the catalytic hydroxyl (or thiol of a cysteine-bearing thioesterase domain) with an amine that is sufficiently reactive to form an amide acyl-enzyme intermediate that fails to react further. This elegant approach allowed for the determination of a structure of the thioesterase domain of the valinomycin NRPS covalently bound to both tetradepsipeptide and dodecadepsipeptide intermediates although only the residues closest to the serine nucleophile were ordered within the crystal lattice. The discovery of tools for modifying the protein with DAP or the substrate with a FP mimic will enable the determination of more covalent acyl-enzyme intermediates and further enrich our understanding of these important enzymes.

Experiments with seryl peptides and corresponding peptides bearing a β-lactam ring have shown exquisite discrimination by NocTE. This specificity likely ensures that β-lactam formation has properly occurred prior to epimerization and hydrolysis within the NocTE active site. The nocardicin ligand mimic allowed us to capture the NocTE protein with high quality electron density to model the complete ligand. This observation reflects the fidelity with which the phosphonate models the ester linkage of the native substrate and captures the avidity of an enzyme for its transition state through multiple points of contact. Additionally, many structurally characterized TE domains catalyze macrocyclization, and not hydrolysis, of their acyl-enzyme intermediate. Structural evidence points to wide active sites within these enzymes that guide intramolecular attack to form the macrolactone product but apparently do not precisely hold the ligand in a single, well-ordered conformation. In contrast, the hydrolysis of the NocTE reaction following the prior epimerization partial reaction, may demand a more traditional active site in which the substrate is bound in an extended conformation with numerous protein-substrate interactions. The on-going discovery of the expanded catalytic roles by NRPS domains^4^ demonstrates that there is much to learn about their ability to produce novel natural products.

## Methods

### NocTE expression and purification

Recombinant His_6_-tagged wild-type NocTE was prepared as described previously^17^. The NocTE protein construct included residues Val1690–Arg1925 of the NocB protein (Genbank AAT09805; UNIPROT Q5J1Q6). pET28b plasmid containing the excised *nocTE* gene was electroporated into Rosetta 2 (DE3) cells and plated on LB agar supplemented with 50 μg/mL kanamycin and 50 μg/mL chloramphenicol overnight at 37 °C. Starter cultures (2 × 35 mL) were grown overnight at 37 °C from a single isolated colony in LB broth containing 50 μg/mL kanamycin and 50 μg/mL chloramphenicol. 10 mL of starter culture was added to 6 × 1 liter of 2 × YT medium with 50 μg/mL kanamycin and 50 μg/mL chloramphenicol and allowed to grow to an optical density (A_600_) of 0.6–0.7 at 37 °C, 200 r.p.m. before being cooled to 4 °C over 1–2 h. The cultures were then induced with aqueous IPTG (1 mM) and shaken at 18 °C, overnight at 200 r.p.m. before cells were harvested by centrifugation (4000 × g, 30 min, 4 °C) and stored at −80 °C until purification. Cells were thawed in cold lysis buffer (50 mM Na/PO4, 300 mM NaCl, pH 8.0, 3 mL/g cells) prior to sonication over ice (60% amplitude, 9.9 s on/off, 7 min; Model GEX 400, Cole-Parmer, Vernon Hills, IL). The viscous lysate was cleared by centrifugation (25,000×*g*, 30 min, 4 °C) and the supernatant incubated with 12 mL of 50% TALON cobalt affinity-resin (2 mL/L culture; Clontech Laboratories, Mountain View, CA) for 1–2 h, 4 °C in a batch-binding format. Following pelleting by centrifugation (750×*g*, 5 min, 4 °C), the resin was resuspended in lysis buffer and transferred to a gravity column where it was further washed with lysis buffer supplemented with 20 mM imidazole (2 × 12 mL). NocTE was next eluted from the column with lysis buffer containing 200 mM imidazole (3 × 12 mL). Analysis by SDS-PAGE and Coomassie staining determined adequately pure fractions of NocTE to be pooled (Supplementary Fig. 8). Combined fractions were concentrated by centrifugation (Amicon Ultra 0.5 ML 10 kMWCO filtration device, 4000×*g*, 10 min, 4 °C) to 8 mL before being twice dialyzed against 2 L of 10 mM HEPES, 25 mM NaCl, pH 7.5 at 4 °C. The concentration of isolated NocTE was estimated by Bradford assay.

### Synthesis of inhibitor

The methylfluorophopshonate derivative of nocardicin G (**4**) was prepared as its C-terminal racemate as previously described^28^. Briefly, fluorinative hydrolysis of nocardicin G diphenylphosphonate (**7**) using ammonium fluoride in dry acetonitrile produced the free fluorophoshonate salt, which was subsequently dissolved in anhydrous ampoule d_6_-DMSO prior to methylation with freshly distilled ethereal diazomethane. The FP **4** stock solution was either immediately used to inhibit NocTE or frozen and stored at –80 °C for up to one week.

### Inactivation of NocTE with FP (4)

To a 5.5 mL solution of NocTE (~5.5 mg/mL, ~200 μM, ~ 30 mg in 10 mM HEPES, 25 mM NaCl, pH 7.5) a freshly prepared ~110 mM solution of **4** in anhydrous ampoule d_6_-DMSO was added by 10 μL aliquots over ice. Sequential additions of FP **4** were made until the complete conversion of unmodified NocTE to the inhibitor-complex was determined by intact-protein UPLC-MS (~240 µL total inhibitor volume used). The covalently modified NocTE solution was then frozen as droplets using liquid N_2_ and stored before further purification and crystallization screening.

### Mutagenesis and mutant activity

Site-directed mutants of NocTE were constructed by the overlap extension method^45^, from pET28b/NocTE using the appropriate DNA primers for the desired mutant (Supplementary Table 1 and Supplementary Table 2). PCR reactions were initiated with addition of Herculase II DNA polymerase (New England Biolabs). The extension-overlap PCR products were ligated into linearized pET28b vectors to provide the TE domain mutants. The PCR product was subcloned into pCRBlunt-TOPO and sequence verified. The pCRBlunt-TE mutant constructs were digested with NdeI and HindIII and ligated with T4 DNA ligase into a similarly digested pET28b vector to create an N-terminal His_6_-tagged expression construct.

To test the in vitro activities of NocTE mutant proteins, 1 mM of *epi*-nocardicin G SNAC (**5**) was added to a 300 μL solution containing 20 μM of freshly purified protein in 50 mM KiPO4 buffer, pH 7.5. Reactions were incubated at room temperature for 3 h and acidified to pH 2 with an aqueous solution of TFA. The enzyme was removed with an Amicon Ultra 0.5 ML 10 kMWCO centrifugal filter (EMD Millipore) and the flow-through was directly analyzed by HPLC, monitoring absorption at 272 nm. HPLC analyses of enzymatic reactions were performed on an Agilent model 1200 equipped with a multi-wavelength UV-Vis detector in conjunction with a reverse-phase Phenomenex Luna 5 u phenyl/hexyl analytical column (250 × 4.60 mm ID) using water (solvent A) and acetonitrile with 0.1 % TFA (solvent B). The method followed: 0–5 min isocratic 7% solvent B, 5–22 min gradient 7 to 50% solvent B, 22–25 min gradient 50 to 7% solvent B, 25–35 min isocratic 7 % solvent B. Flow rate was 1.0 mL/min.

### X-ray data collection and structure determination

The structure of the NocB TE domain was initially solved by multiwavelength anomalous dispersion (MAD) phasing with selenomethionine (SeMet) labeled protein. Automated crystal screening was performed at the University of Maryland Crystallography Core using an OryxNano (Douglas Instruments) with ~15 mg/ml *N*-His_6_-NocTE and solutions from Qiagen. Crystallization was repeated using sitting-drop vapor diffusion against a 1 mL reservoir solution of 50 mM Bis-Tris pH 5.5, 2.0 M ammonium sulfate and 2–4 μL drops initially composed of a 1:1 mixture of protein and well solution. Crystallization was repeated with SeMet-labeled protein and cryoprotected by immersion into 50 mM Bis-Tris pH 5.5, 2.2 M ammonium sulfate, and 20% glycerol. Crystals were not stable under these conditions and were quickly cooled using liquid N_2_. Diffraction data for a two-wavelength MAD experiment were collected at the National Synchrotron Light Source on beamline X6A (Supplementary Table 3). Data were indexed, integrated, and scaled using HKL2000^46^, which showed an anomalous signal extending to ~3.0 Å. Experimental phasing with BP3 was followed by density modification with Solomon and automated model building using Buccaneer, as implemented in the CRANK pipeline in CCP4^47–49^. The resulting model showed the anticipated and highly-conserved α/β fold and was ~ 70% complete. Iterative model building in Coot and refinement in Refmac5 against the remote dataset^50,51^ enabled building of the complete thioesterase domain, with several small disordered loops.

To improve crystal quality and potentially find an alternate crystal form, an additional step was incorporated into the purification protocol. Gel filtration chromatography with a S200 16/600 column (GE Healthcare) was used to isolate homogeneous protein and exchange into buffer containing 10 mM HEPES pH 7.5, 25 mM NaCl, 0.2 mM TCEP. Subsequently, clusters of crystals of unliganded NocTE were obtained in two to six days at 15 mg/ml by sitting drop vapor diffusion with 50 mM Bis-Tris pH 5.0 and 2.0 M ammonium sulfate at 20 °C. Further optimization of the crystallization condition by varying Bis-Tris pH 5.0–5.5 and drop volume ratio led to growth of hexagonal single crystals. Cryoprotection of crystals prior to flash-freezing in liquid nitrogen was done by serial transfer into mother liquor containing 5, 10, and 20% glycerol in the presence of 1 mM fluorophosphonate inhibitor **4** in the hope of covalently modifying the protein during the cryoprotection procedure. Data collection from a single crystal was performed remotely at beamline 23ID-B, GM/CA-CAT, Advanced Photon Source and processed using XDS. Data were processed to 1.8 Å resolution in the *P*321 space group (Supplementary Table 3). Molecular replacement was performed with Phaser using coordinates of the initial SeMet model as the search model^52^. The resulting model containing one protein molecule per asymmetric unit was further refined iteratively using COOT and PHENIX.refine with TLS parameters. Despite the presence of the fluorophosphonate ligand in the cryoprotection solutions, no density was observed for ligand bound to the catalytic serine.

We, therefore, identified new crystallization conditions with protein covalently modified by preincubation with the FP ligand **4**. New conditions were identified by sitting drop vapor diffusion using a cocktail composed of 50 mM CHES pH 9.0, 250 mM CaCl_2_, 30% PEG 4000. Thin plate-like crystals obtained initially were used as seeds for further optimization in the same condition using a drop of 1 µL of protein + 1 µL of crystallization cocktail in MRC 3 sub-well plates at 20 °C. High quality crystals were observed in three to six days. Crystals were cryoprotected by serial transfer into mother liquor containing 8, 16, and 24% glycerol. Data collection from single crystals was performed remotely at APS beamline 23 ID-D. Diffraction data were processed using XDS to a resolution of 2.0 Å in space group *P*2_1_2_1_2_1_. Protein atoms from the unliganded NocTE structure were used as the initial search model for molecular replacement with Phaser. A solution with four protein molecules in asymmetric unit was obtained. Model building was done using COOT followed by refinement using PHENIX.refine. Final model building and refinement with ligand was done using COOT and REFMAC5 with NCS restrains and jelly-body refinement.

### Reporting summary

Further information on research design is available in the Nature Research Reporting Summary linked to this article.

## Supplementary information

Supplementary Information

Reporting Summary

## Data Availability

The coordinates and structure factors for the two structures are available from the Protein Data Bank for unliganded NocTE, accession 6OJC, and bound to the fluorophosphonate inhibitor, accession 6OJD. Other data are available from the corresponding authors upon reasonable request.
