# Supplementary material for: Structure of a bound peptide phosphonate reveals the mechanism of nocardicin bifunctional thioesterase epimerase-hydrolase half-reactions
Source: Nat Commun. 2019 Aug 27;10:3868. doi: 10.1038/s41467-019-11740-6 (PMC6711958; doi:10.1038/s41467-019-11740-6)
Supplement: Supplementary file 1 — Supplementary Information [file 41467_2019_11740_MOESM1_ESM.pdf]

Supplementary Information

Structure of a Bound Peptide Phosphonate Reveals the Mechanism of Nocardicin Bifunctional Thioesterase Epimerase-Hydrolase Half-Reactions

Ketan D. Patel, Felipe B. d'Andrea, et al.

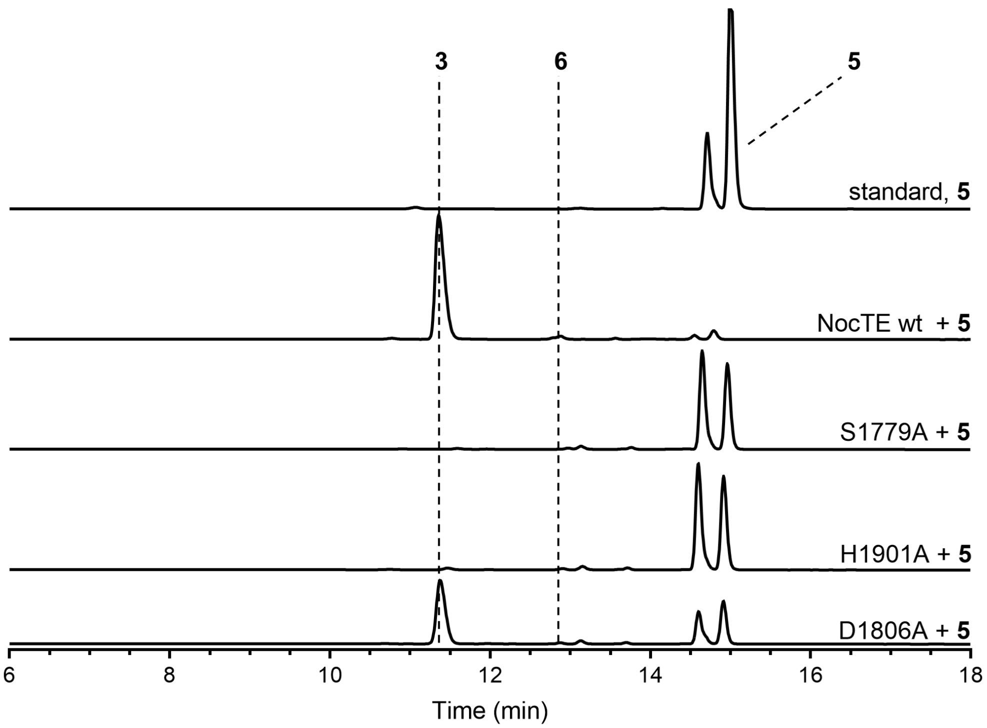

Supplementary Figure 1. Mutational analysis of NocTE catalytic triad residues. Reactions of the SNAC thioester of *epi*-nocardicin G (**5**) with NocTE active site mutants S1779A, H1901A, and D1806A were analyzed by HPLC along with a wild-type control, which produced nocardicin G (**3**) and trace amounts of *epi*-nocardicin G (**6**). The S1779A and H1901A mutants were completely inactive leaving an equilibrium mixture of substrate **5** and its *C*-terminal epimer by comparatively slow chemical exchange. The D1806A mutant was modestly active under the conditions of the assay, but stereochemically faithful to the wild type reaction.

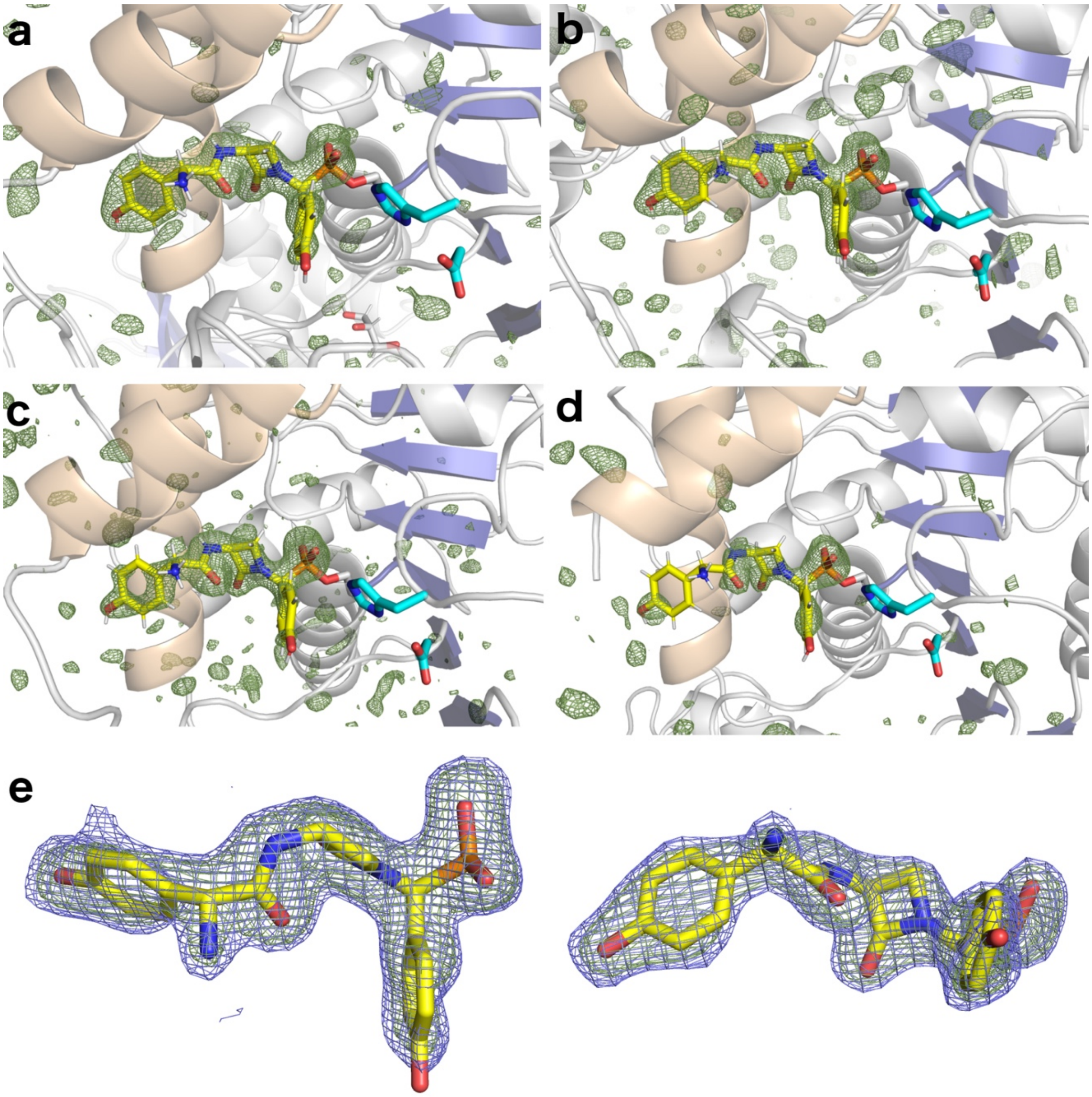

Supplementary Figure 2. Electron density of covalent phosphonate ligand. Simulated annealing omit map electron density (top), contoured at 3 σ for the ligand in all four chains. **a**. Chain A, **b**. Chain B, **c**. Chain C, and **d**. Chain D. Density is shown without a carve radius to provide an accurate view of density quality. **e**. Orthogonal orientations of the refined electron density with coefficients of the form 2*Fo*-*Fc* is shown in blue, along with the simulated annealing electron density (green) for peptide ligand of chain A.

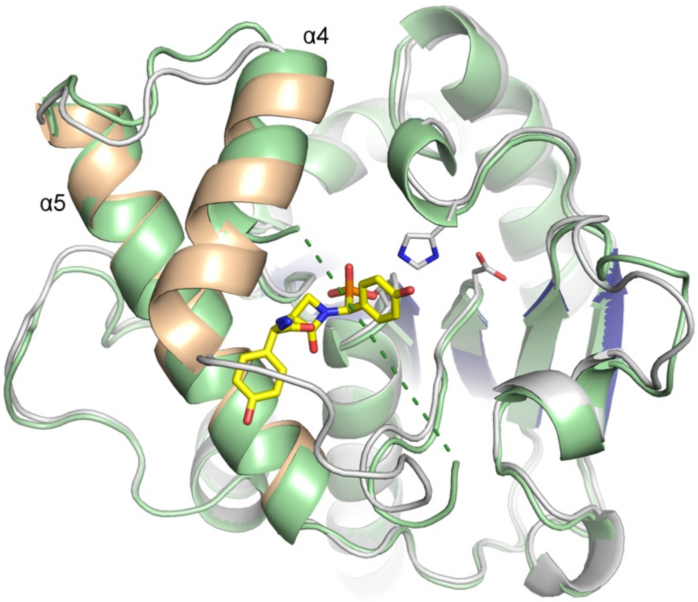

Supplementary Figure 3. Superposition of unliganded and peptide-bound NocTE. The peptide bound structure is shown with grey ribbon, with the central β-sheet highlighted in blue and the two lid α-helices shown in wheat. The structure of unliganded NocTE is shown in light green. The dashed line represents the disordered region from the unliganded structure between residues Ala1813 and Gly1822. This loop, along with the N-terminal region of helix α4 becomes ordered in the peptide bound structure.

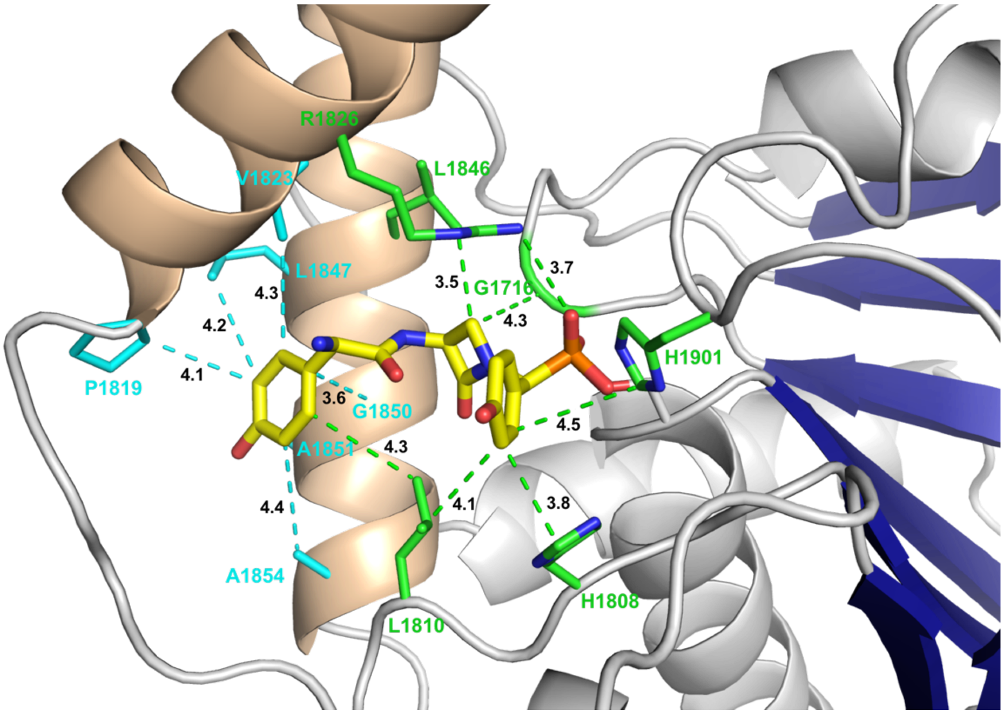

Supplementary Figure 4. NocTE residues that interact with the phosphonate ligand. Residues highlighted in cyan are part of the hydrophobic groove that interacts with *N*-terminal pHpg group of ligand. Residues in green are interacting with the β-lactam and *C*-terminal pHpg.

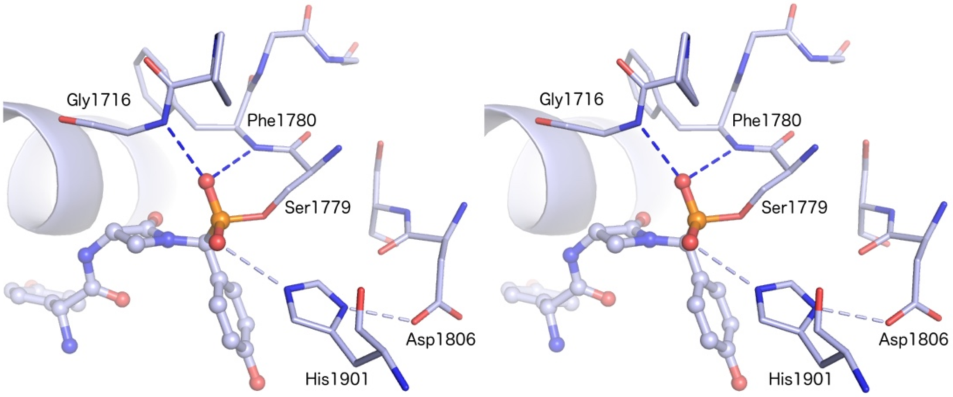

Supplementary Figure 5. Illustration of the phosphonate ligand in the oxyanion hole. Stereorepresentation of the oxyanion hole depicting the ligand of chain A bound covalently to Ser1779. One oxygen of the phosphonate moiety interacts with catalytic triad residue His1901 while the other interacts with the amide nitrogens of Gly1716 and Phe1780, which form the oxyanion hole.

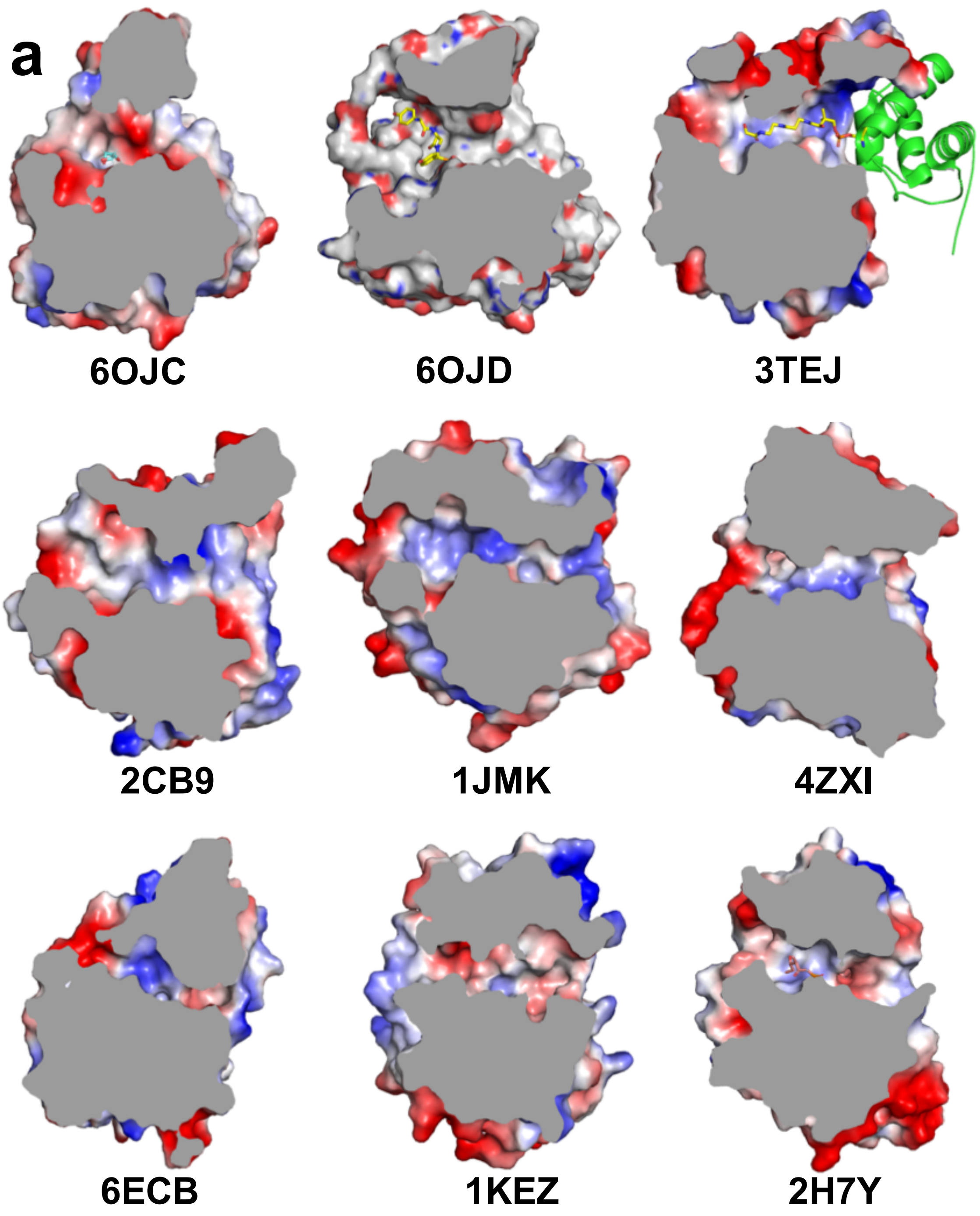
a
6OJC
6OJD
3TEJ
2CB9
1JMK
4ZXI
6ECB
1KEZ
2H7Y

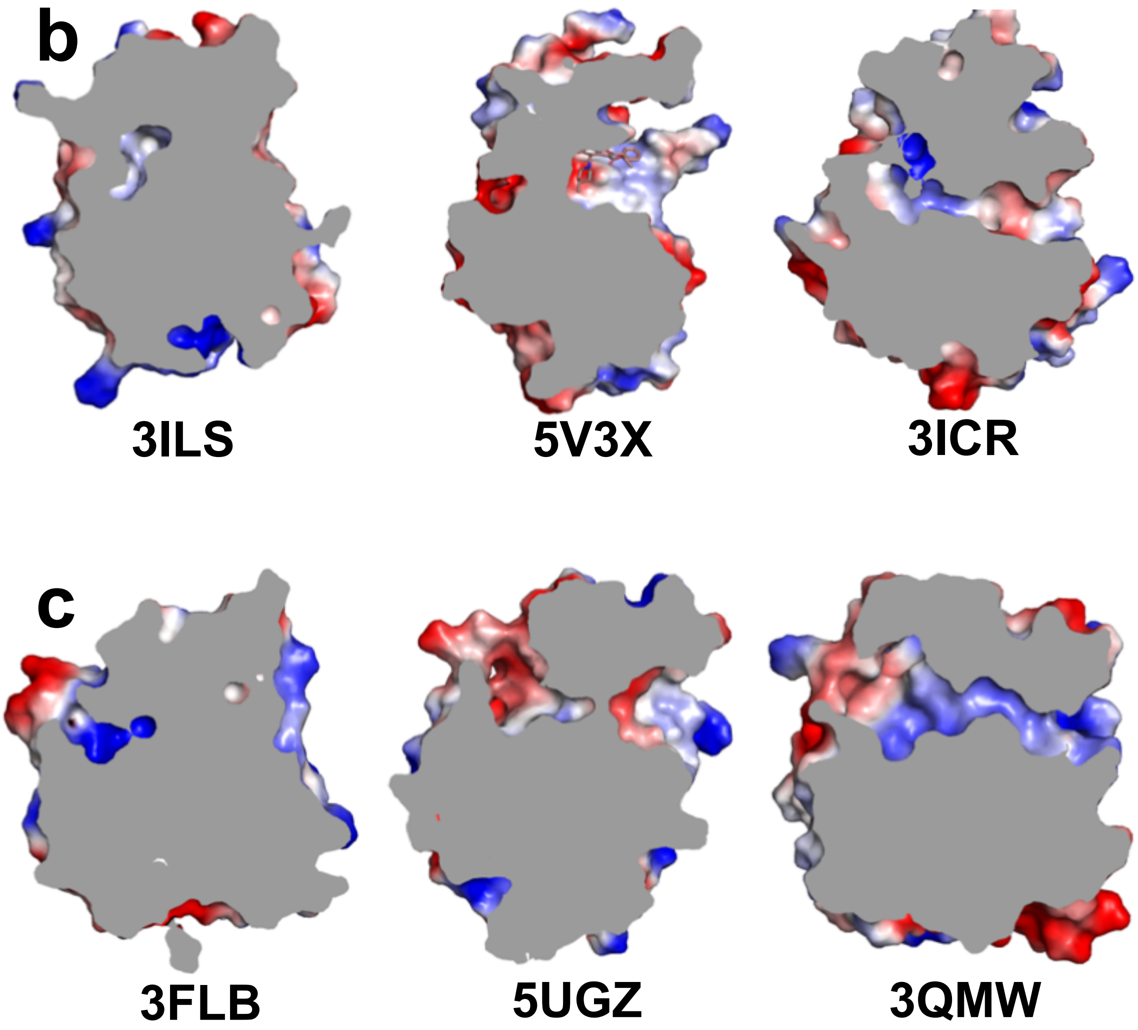

Supplementary Figure 6. Electrostatic surface representation of substrate binding channel in NRPS/PKS TE domains as observed in crystal structures. **a**. Open channel in unliganded NocTE (6OJC), NocTE complex (6OJD), EntF-PCP (3TEJ), FengTE (2CB9), SrfTE (1JMK), AB3403TE (4ZXI), Vlm2TE (6ECB), DEBSTE (1KEZ) , and PikTE (2H7Y). **b**. Closed channel in PksATE (3ILS), Pks13TE (5V3X) and Tautomycetin TE (3ICR). **c**. Type-II TE domains show closed channel in RifR (3FLB), open from two ends but blocked at middle in Colibactin_TE, ClbQ (5UGZ) and two open ends in RedJ (3QMW). All representations are aligned to EntF_TE (3TEJ) with PCP-binding site towards right side as shown in panel **a**.

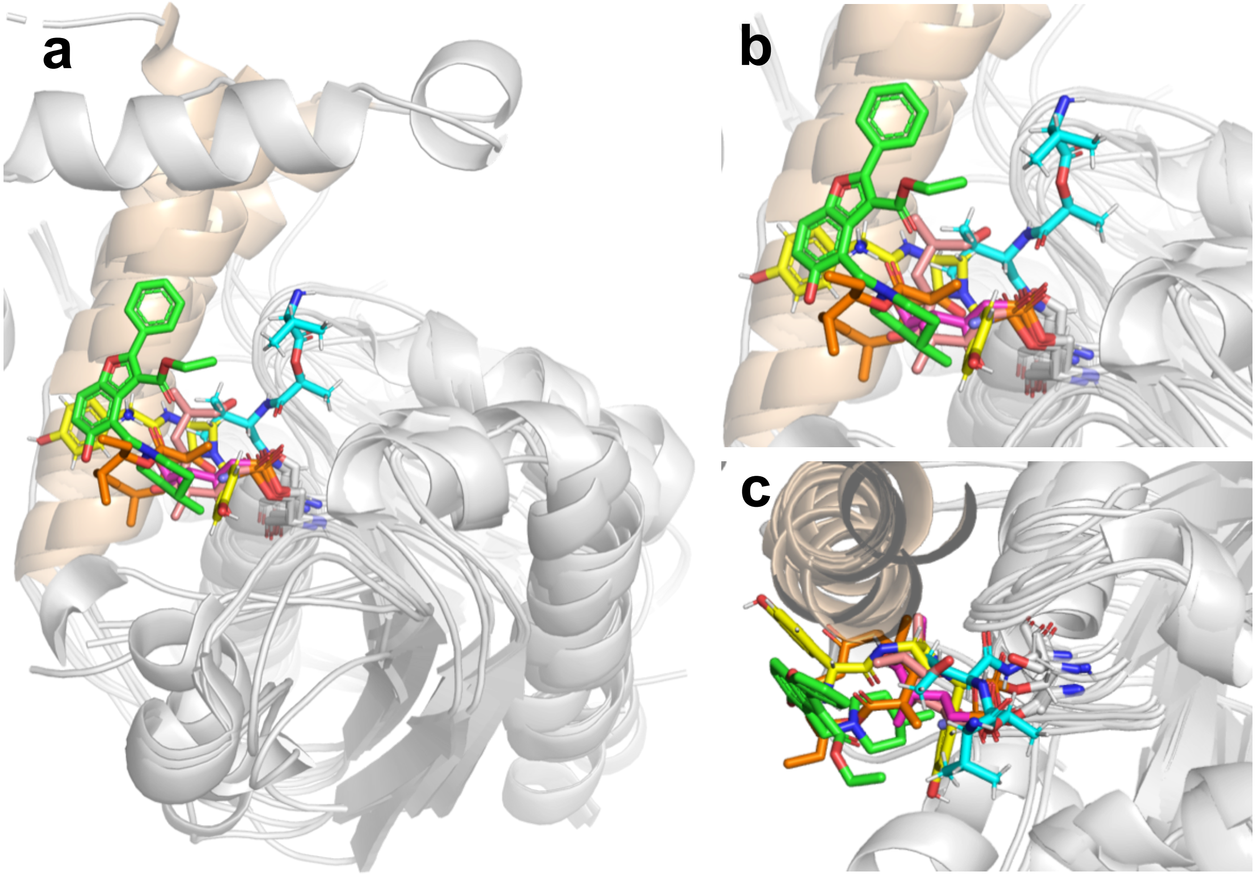

Supplementary Figure 7. Orientation of ligand binding in NRPS and PKS TE domains. **a**. Ligands projecting towards corresponding lid helix α5 of NocTE (highlighted in wheat). Core N-terminal domain and other lid helices are colored grey, loops are smoothed for clarity. Loops and helices from several structures are deleted to emphasize the orientation of the ligands. Carbon atoms of ligands are colored yellow for NocTE-complex (6OJD), light blue for DEBS-TE (5D3Z), magenta, salmon and orange for PikTE (2H7X, 2HFJ, 2HFK respectively), green for Pks13 (5V3X), and cyan for Valinomycin TE, Vlm2 (6ECE). A close-up view is shown in **b** and **c**, which is rotated by ~90°.

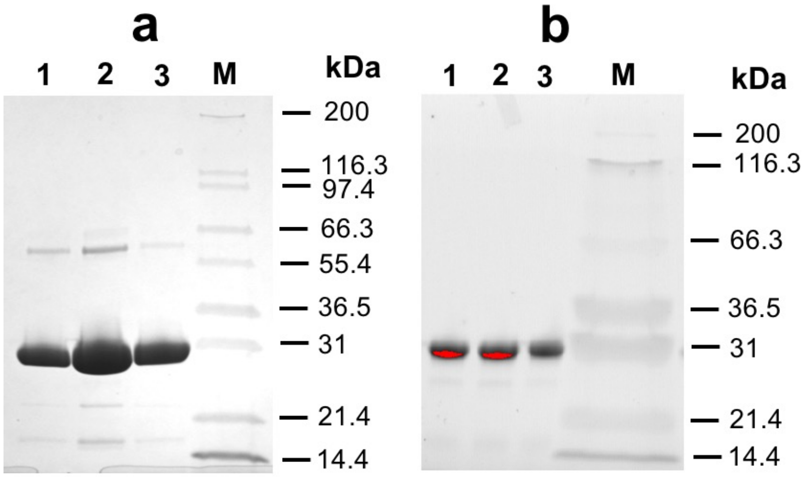

Supplementary Figure 8. SDS-PAGE of purified NocTE. **a**. Lanes 1-3: Unliganded NocTE protein; M, Mark12 protein marker. **b**. Lanes 1-3: Covalently inactivated NocTE protein; M, Mark12 protein marker.

Supplementary Table 1. Oligonucleotides Sequences

Primer Name	Nucleotide Sequence[a]
NocTE-NdeI	5’-GGGATACATATGGTCGAGGGCTCCGGG-3
NocTE-HindIII	5’-GGATAAAGCTTTCACCGCTCTCCTCCCAG-3’
S1779A-F	5’-CGGCGGCTGG**GCC**TTCGGCGGCG-3
S1779A-R	5’-CGCCGCCGAA**GGC**CCAGCCGCCG-3’
D1806A-F	5’-CTGCTGCTCGTC**GCC**AGCCACAACCTC-3’
D1806A-R	5’-GAGGTTGTGGCT**GGC**GACGAGCAGCAG-3’
H1808A-F	5’-GTCGACAGC**GCC**AACCTCAACGCC-3’
H1808A-R	5’-GGCGTTGAGGTT**GGC**GCTGTCGAC-3’
H1808Q-F	5’-GTCGACAGC**CAG**AACCTCAACGCC-3’
H1808Q-R	5’-GGCGTTGAGGTT**CTG**GCTGTCGAC-3’
H1808N-F	5’-GTCGACAGC**AAC**AACCTCAACGCC-3’
H1808N-R	5’-GGCGTTGAGGTT**GTT**GCTGTCGAC-3’
H1901A-F	5’-GTGCCGGGCGCG**GCC**GAGCGGTTGTTC-3’
H1901A-R	5’-GAACAACCGCTC**GGC**CGCGCCCGGCAC-3’

[a]Restriction sites are underlined and mutation codon is highlighted in bold.

Supplementary Table 2. Oligonucleotides for Mutagenesis

Construct	5’ Primer	3’ Primer
NocTE S1779A	NocTE-Nde1	S1779A-R
	S1779A-F	NocTE-HindIII
NocTE D1806A	NocTE-Nde1	D1806A-R
	D1806A-F	NocTE-HindIII
NocTE H1808A	NocTE-Nde1	H1808A-R
	H1808A-F	NocTE-HindIII
NocTE H1808Q	NocTE-Nde1	H1808Q-R
	H1808Q-F	NocTE-HindIII
NocTE H1808N	NocTE-Nde1	H1808N-R
	H1808N-F	NocTE-HindIII
NocTE H1901A	NocTE-Nde1	H1901A-R
	H1901A-F	NocTE-HindIII

Supplementary Table 3. Crystallographic data

	SeMet (Peak)	SeMet (Remote)	Native	Liganded
**PDB ID**			**6OJC**	**6OJD**
**Data Collection**				
Resolution (Å)[a]	50-2.2 (2.25-2.2)	50-2.2 (2.25-2.2)	29-1.79 (1.83-1.79)	40-1.99 (2.06-1.99)
Space group	*P*321	*P*321	*P*321	$P2_12_12_1$
Unit cell a, b, c (Å)	113.6, 113.6, 46.3	113.6, 113.6, 46.3	114.7, 114.7,46.6	73.7, 78.6, 146.9
α, β, γ (°)	90, 90, 120	90, 90, 120	90, 90, 120	90, 90, 90
Total Observations	188493	183756	313022	109284
Unique reflections			33409	58015
Multiplicity	10.7 (8.4)	10.5 (8.2)	9.4 (9.3)	3.2 (3.3)
Completeness (%)	99.5 (95.5)	99.0 (90.7)	99.7 (97.2)	98.3 (98.5)
Mean I/sigma(I)	35.9 (3.7)	35.3 (4.2)	25.6 (1.0)	10.1 (2.4)
R_{MERGE}	5.7 (27.1)	5.2 (28.2)	0.05 (1.6)	0.04 (0.38)
R_{MEAS}			0.05 (1.7)	0.06 (0.53)
CC1/2			0.99 (0.52)	0.99 (0.78)
**Structure Refinement**				
Refinement Resolution			29-1.94 (2.02-1.94)	40-1.99 (2.06-1.99)
R_{WORK} (%)			20.65 (29.56)	19.12 (30.93)
R_{FREE} (%)			22.99 (32.41)	22.33 (35.12)
Number of non-hydrogen atoms				
protein			1690	6582
ligand			36	188
solvent			91	393
RMSD bond len (Å)			0.017	0.009
RMSD bond angles (°)			1.54	1.28
Ramachandran analysis				
favored (%)			97.7	97.2
allowed (%)			2.3	2.7
outliers (%)			0	0.1
Rotamer outliers (%)			1.2	0

[a]Values in parentheses are for the highest resolution shell.
